# 90 YEARS OF PROGESTERONE: Selective progesterone receptor modulators in gynaecological therapies

**DOI:** 10.1530/JME-19-0238

**Published:** 2020-01-30

**Authors:** H O D Critchley, R R Chodankar

**Affiliations:** 1 MRC Centre for Reproductive Health, The University of Edinburgh, The Queen’s Medical Research Institute, Edinburgh Bioquarter, Edinburgh, UK

**Keywords:** abnormal uterine bleeding (AUB), heavy menstrual bleeding (HMB), selective progesterone receptor modulators (SPRM), leiomyoma, fibroid

## Abstract

Abnormal uterine bleeding (AUB) is a chronic, debilitating and common condition affecting one in four women of reproductive age. Current treatments (conservative, medical and surgical) may be unsuitable, poorly tolerated or may result in loss of fertility. Selective progesterone receptor modulators (SPRMs) influence progesterone-regulated pathways, a hormone critical to female reproductive health and disease; therefore, SPRMs hold great potential in fulfilling an unmet need in managing gynaecological disorders. SPRMs in current clinical use include RU486 (mifepristone), which is licensed for pregnancy interruption, and CDB-2914 (ulipristal acetate), licensed for managing AUB in women with leiomyomas and in a higher dose as an emergency contraceptive. In this article, we explore the clinical journey of SPRMs and the need for further interrogation of this class of drugs with the ultimate goal of improving women’s quality of life.

## Introduction

Selective Progesterone Receptor Modulators (SPRMs) are a class of synthetic steroids with different molecular structures. They interact with the progesterone receptor (PR) and may exert an agonist, antagonist or a mixed response (Lusher *et al*. 2011). Progesterone plays a vital role in the structure, function and regulation of the female reproductive tract, including pregnancy. Progesterone mediates its function by interacting with the PR, a member of a superfamily of almost 50 ligand-activated nuclear transcription factors (McEwan 2009).

A large number of gynaecological problems such as abnormal uterine bleeding (AUB), fibroids (leiomyoma), adenomyosis, endometriosis and reproductive tract cancers are hormonally mediated; therefore, SPRMs hold great potential for the management of women with gynaecological disorders.

## History of SPRMs

The search for drugs that modify progesterone activity with an aim to achieve contraception can be traced back to the 1960s (Pincus 1960). The first SPRM, RU486 (mifepristone), was discovered in the 1980s, during the quest for discovery for anti-glucocorticoid drugs (Moguilewsky & Philibert 1984).

**Figure 1:**
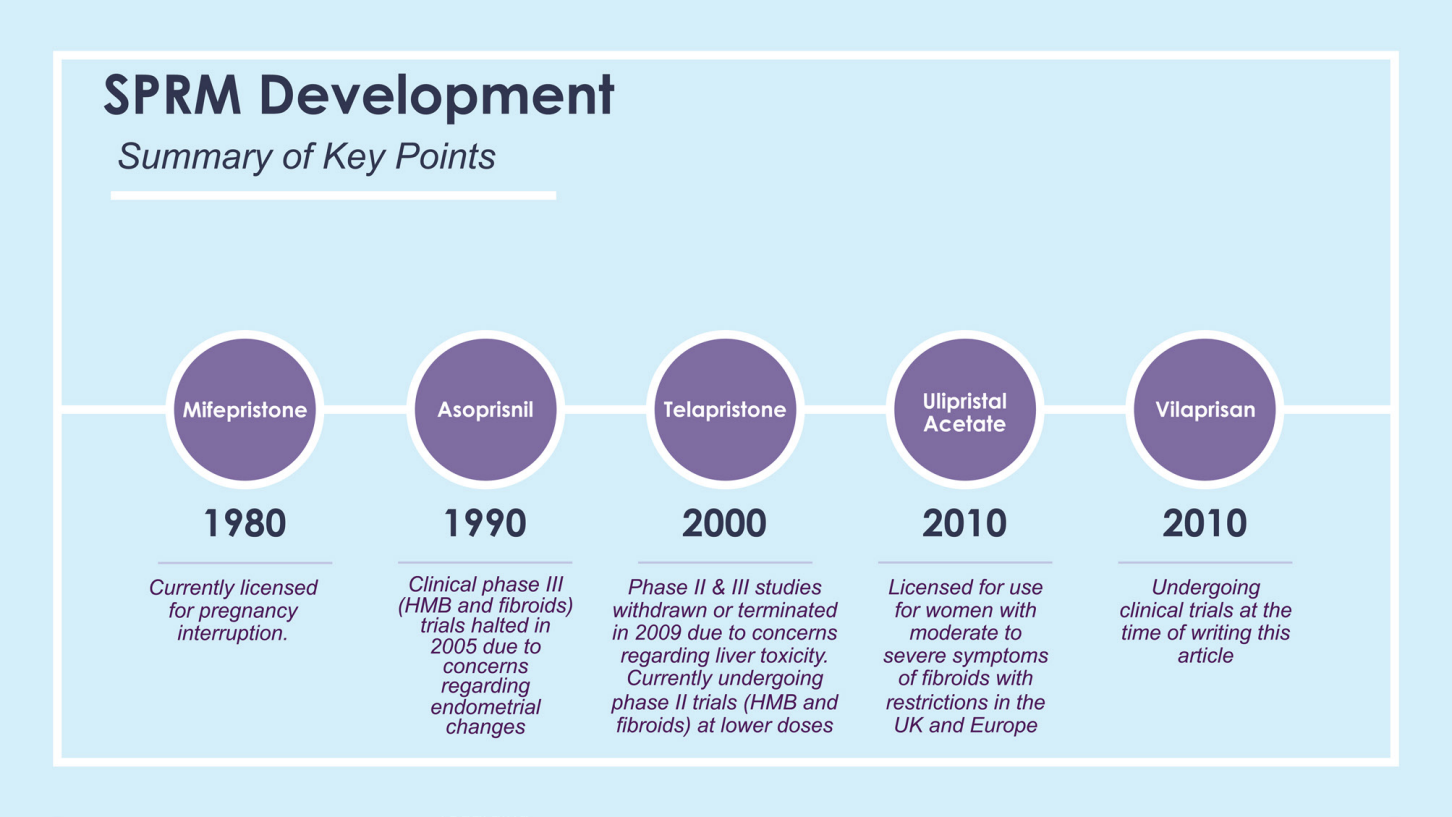
Key points in the of the development of SPRMs (timeline) and current clinical significance. Mifepristone and ulipristal acetate are the only SPRMs in current clinical use. The use of Asoprisnil was halted due to concerns regarding endometrial changes (Guo & Groothuis 2018, Lewis *et al*. 2018). Telapristone studies were halted due to liver toxicity concerns (Lewis *et al*. 2018).

Several SPRMs have been developed (Fig. 1) since, and the latest in this class of drugs is vilaprisan. The development of SPRMS is shown in the timeline in Fig. 1.

Non-steroidal SPRMS aim to achieve the effect of progesterone receptor binding that can selectively act as a progestin in the endometrium while acting as an antiprogestin within the leiomyoma; however, there is a limited clinical translation of these agents (Catherino *et al*. 2010).

## Clinical need for SPRMs

Heavy Menstrual Bleeding (HMB) affects one in four women of reproductive age. It accounts for over a million annual referrals to the gynaecology services in the United Kingdom (UK) (Shapley *et al*. 2004, Royal College of Obstetricians and Gynaecologists 2014). The effects of HMB may be so profound that the National Institute for Clinical Excellence (NICE 2018) defines HMB as any bleeding that negatively impacts the woman’s physical, emotional, social and/or material quality of life (NICE 2018). This definition steers us away from the traditional definition (now only used in a research context – menstrual blood loss over 80 mL/cycle) of quantitatively estimating blood loss to define HMB (Hallberg & Nilsson 1964, Warner *et al*. 2004).

HMB is also associated with economic implications for the healthcare system and loss of productivity due to time off work and presenteeism. Medical treatments may be ineffective, unsuitable or have undesirable side effects for some women. Surgical treatments for HMB may be invasive and may have associated surgical and anaesthetic risks and may cause a permanent loss of fertility for women (Frick *et al*. 2009).

A recent review using the data derived from the National HMB Audit (England and Wales) included nearly 15,000 women. The data showed that 54% of women seeking HMB treatment were under 45 years, with approximately two-thirds having no other co-morbidity. Half of the women under 45 years received fertility-ending surgery (hysterectomy or endometrial ablation) in the first year of referral to secondary services (Geary *et al*. 2019). However, given over half of all UK-born babies (55%) are to women aged 30 or older, fertility-ending surgery is not always acceptable (Office for National Statistics 2017). A recent Dutch study based on an internet survey of nearly 43,000 women suggested that because of menstrual symptoms, nearly 38% of women reported being unable to perform their regular daily activities (Schoep *et al*. 2019). There remains an unmet clinical need in managing women with HMB.

The role of SPRMs in emergency contraception is well established. Further exploration is ongoing to investigate the role of SPRMs as an oestrogen-free method of long-term contraception.

This article focuses on the SPRMS in current clinical use or undergoing investigation in clinical trials (Fig. 1).

## Ulipristal acetate (UPA or CDB-2914 or VA-2914)

**Figure 2:**
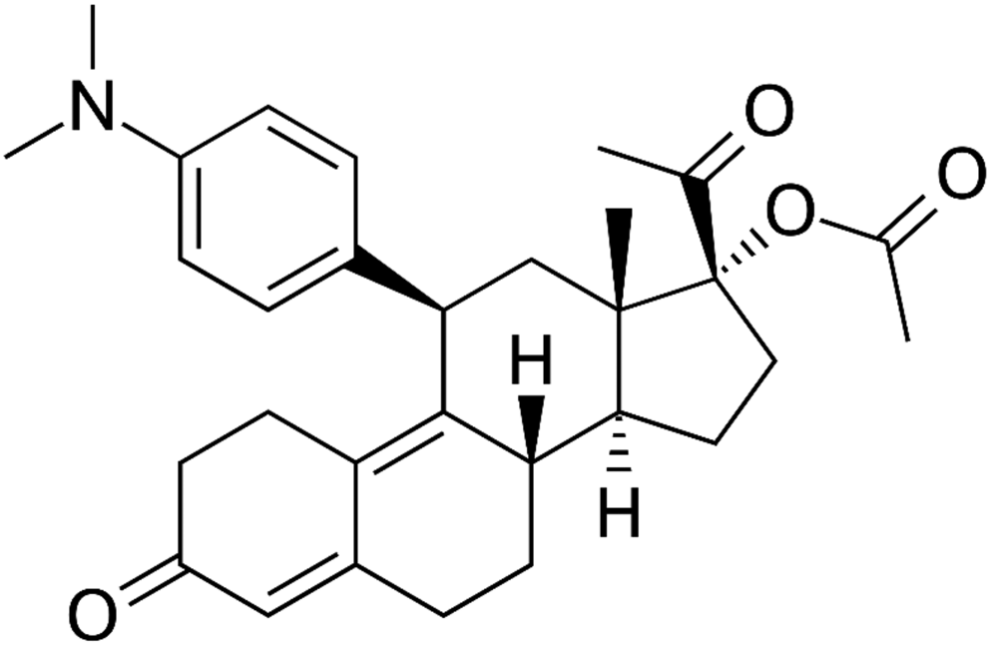
Chemical structure of UPA.

UPA was first studied in the 1990s in the context of an ‘antifertility’ drug in keeping with the properties of RU486 (mifepristone), both in rats and humans (Passaro *et al*. 1997, Reel *et al*. 1998). Like mifepristone, UPA was labelled an ‘antiprogestin’ when initially developed and only in recent years has been classed as an SPRM. UPA is a steroidal SPRM with a structure of a 19 norprogesterone derivative: 17a–Acetoxy-11b-(4-N, N-Dimethylaminophenyl)-19-norpregna-4-9-diene-3,20 dione, also known as CDB 2914, since it was initially developed by the National Institute of Child Health and Human Development (NICHD). It is also known as HRP 2000 or VA 2914 (Bouchard 2014). The chemical structure of UPA is illustrated in Fig. 2.

## Emergency Contraception (EC) Overview (United Kingdom)

UPA is United States Food and Drug Administration (US FDA) approved as an emergency contraceptive and is licensed in the United Kingdom for this purpose, including for over the counter (OTC) use (European Consortium for Emergency Contraception 2017). Evidence suggests that the most effective emergency contraceptive is a Copper intrauterine device (Cu-IUD). It has a failure rate of <1% when inserted within 5 days (120 h) after the first unprotected sexual intercourse (UPSI) in a natural cycle or within 5 days after the earliest estimated date of ovulation (whichever is later). The Faculty of Sexual and Reproductive Healthcare (FSRH) suggests it has the added advantage of providing ongoing contraception (Cleland *et al*. 2012, The Faculty of Sexual & Reproductive Healthcare 2017). There are limitations of using an IUD for this purpose; it cannot be used in women with an active pelvic infection, undiagnosed genital tract bleeding or distortion of uterine anatomy. It requires a medical professional available for insertion. Ongoing risks include that of uterine perforation, abnormal uterine bleeding and dysmenorrhoea in some users.

Other emergency contraceptive methods licensed in the United Kingdom include oral levonorgestrel (LNG) and oral UPA. LNG is used in a dose of 1.5 mg orally (single dose) and is licensed for use up to 72 h after UPSI or contraceptive failure. UPA is used in a dose of 30 mg and is licensed for up to 120 h for the same indications (The Faculty of Sexual & Reproductive Healthcare 2017). Current evidence suggests that UPA is more effective than LNG as an emergency contraceptive (Glasier *et al*. 2010, Shen *et al*. 2019).

The combined hormonal ‘Yuzpe method’ is no longer recommended for use in the United Kingdom, as evidence suggests lower efficacy as compared to LNG EC alone (Cheng *et al*. 2012, Leung *et al*. 2016). The oestrogen–progestin regimen comprises two doses of a combination of 100 μg of ethinyl oestradiol and 0.5 mg of levonorgestrel each, the first dose taken within 72 h after intercourse and the second 12 h later (Yuzpe & Lancee 1977, Glasier 1997). There is a current lack of evidence to recommend the LNG-IUS as a method of emergency contraception (The Faculty of Sexual & Reproductive Healthcare 2017).

## UPA and Emergency Contraception (EC)

The predominant mechanism of action of UPA is inhibition or delay of ovulation by interrupting the luteinising hormone (LH) surge (Stratton *et al*. 2000). However, even when the LH surge has commenced, UPA can prevent ovulation, suggesting a direct effect on the growing ovarian follicle (Nallasamy *et al*. 2013).

In addition to inhibition of ovulation, endometrial effects (molecular) of UPA are also proposed, which may impact decidualisation and consequently implantation (Lira-Albarran *et al*. 2018). Endometrial effects have also been proposed at a macroscopic level (Stratton *et al*. 2000, 2010, Passaro *et al*. 2003). Hence, UPA may be both contraceptive and contragestive in its actions, making it an effective emergency contraceptive (Keenan 2011).

This effect has been questioned in more recent reviews. Authors propose that the endometrial effects seen with UPA use may simply reflect a consequence of a delay in ovulation or require fairly large doses of UPA which are not currently used for UPA-EC (Li *et al*. 2019).

## UPA and long-term contraception

Given the beneficial effects of UPA as an EC, its role as a long-term contraceptive has been explored in the form of a contraceptive vaginal ring (Jensen 2013). The study suggested that at a dose of 2500 µg/day, ovulation could be suppressed in up to 86% of treatment cycles assessed by transvaginal ultrasound and a hormonal assay. Progesterone receptor modulator associated endometrial changes (PAEC) were seen in nearly 79% of participants; however, PAEC were resolved upon UPA discontinuation (Huang *et al*. 2014). Further investigation is needed to elucidate the role of UPA as a long-term oestrogen free contraceptive.

## UPA and fibroids (leiomyomas)

UPA is the only SPRM specifically approved and commercialised to date for management of symptomatic uterine fibroids. UPA controls HMB in over 90% of women, with an overall decrease in bleeding similar to Gonadotrophin releasing hormone (GnRH) agonist use. It has a faster onset of amenorrhoea, usually within 10 days. Oestradiol levels are maintained in the mid-follicular phase range during treatment, thereby reducing the likelihood of menopausal symptoms. Revised European Medicines Agency (EMA) and Medicines and Healthcare Products Regulatory Agency (MHRA) guidance should be used to guide UPA use (Table 1).

**Table 1:** Current indications and restrictions of use of UPA in the United Kingdom and Europe (adapted from European Medicines Agency 2018, Medicines and Healthcare Products Regulatory Agency 2018).

Indications	Liver function monitoring
UPA is indicated for the intermittent treatment of moderate to severe symptoms of uterine fibroids in women of reproductive age who are not eligible for surgery	Before initiation of each treatment course: perform liver function tests; do not initiate UPA in women with baseline alanine transaminase (ALT) or aspartate aminotransferase (AST) more that two times the upper limit of normal (ULN).
UPA is indicated for one course of preoperative treatment of moderate to severe symptoms of uterine fibroids in adult women of reproductive age	During the first two UPA treatment courses: perform liver function tests every month.
UPA treatment is to be initiated and supervised by a physician experienced in the diagnosis and treatment of uterine fibroids	For further treatment courses: perform liver function tests once before each new course and when clinically indicated.
UPA is contraindicated in women with underlying liver disorders	At the end of each treatment course: perform liver function tests after 2–4 weeks. Stop UPA treatment and closely monitor women with ALT or AST more than three times the upper limit of normal; consider the need for specialist hepatology referral.

## UPA and liver function

UPA was licensed for use in the UK and EU in 2012 for the management of fibroids related HMB. During post-marketing surveillance of women exposed to UPA (approximately 765,000), eight cases of serious liver injury were identified, and of these cases, four required a liver transplant (https://www.ema.europa.eu/en/documents/variation-report/esmya-h-c-2041-a20-0043-epar-assessment-report-article-20_en.pdf last accessed: 08/08/2018). This meant the use of UPA was temporarily halted for investigation. After considering all the evidence, in May 2018, the Pharmacovigilance Risk Assessment Committee (PRAC) of the EMA concluded that UPA may have contributed to the development of some cases of serious liver injury; however, the status of UPA as a medication responsible for drug-induced liver injury (DILI) was not fully confirmed (European Medicines Agency 2018). UPA was reintroduced with clinical restrictions and liver function monitoring (Table 1).

UPA does not belong to any of the drug classes commonly considered as drug-induced liver injury agents, nor has it any molecular features similar to other drugs in the DILI network (Donnez *et al*. 2018*a*). The current understanding is that UPA may be responsible for idiosyncratic (rather than intrinsic) DILI and that the use of liver health monitoring will help to minimise risks associated with its use (Donnez 2018).

From the clinician’s perspective, review of the available clinical trial data suggests there were no cases in the 5 mg once daily (OD) UPA group (approved clinical dose) showing any liver enzymes outside accepted ranges (Donnez 2018, Donnez *et al*. 2018*a*).

Abnormalities in liver function tests have also been previously noted with the use of SPRMs including mifepristone and proellex (CDB-4124). All of these compounds have the 4-(dimethylamino) phenyl group in common. This structural element is accessible to metabolic demethylations, which may lead to the formation of aniline metabolites that have been reported to cause undesired effects in the liver by the formation of reactive intermediates (Lu *et al*. 2015). The data from clinical studies with SPRMs lacking the dimethylamino phenyl group, for example, lonaprisan, have provided no evidence of a clinically relevant, drug-related change in liver enzyme activity (Möller *et al*. 2018).

## UPA and clinical trials

The majority of the clinical evidence for use for UPA in management of women with heavy menstrual bleeding and fibroids is available from the PGL4001 (ulipristal acetate) Efficacy Assessment in Reduction of symptoms due to uterine Leiomyomata, PEARL (four phase three trials; PEARL I–IV), and Assessment of Endometrial Safety During Treatment of Symptomatic Uterine Fibroids With Ulipristal Acetate, VENUS (I–II) trials (Donnez *et al*. 2012*a*,*b*, 2014, 2015, Illingworth *et al*. 2018, Simon *et al*. 2018) (Table 2).

**Table 2:** Summary of key findings of phase III clinical trials utilising UPA for management of HMB and fibroids.

Study	Type	Population	Intervention and comparator	Outcomes	Conclusion
PEARL I	Randomised Double blind Placebo controlled	Women with symptomatic fibroids; Age: 18–50 years; BMI: 18–40 kg/m^2^; PBAC score >100 (first 8 days of menstruation); At least one uterine fibroid >3 cm but <10 cm; Fibroid uterus: <6 weeks; Anaemia: Hb <10.2 g/dL; Eligible for surgery	Women were randomised in a ratio of 2:2:1 to 5 mg/day UPA (*n* = 96) 10 mg/day UPA (*n* = 98) Placebo (*n* = 48) Treatment duration 12–13 weeks All women received 80 mg/day oral iron	PBAC score <75 was achieved in 5 mg UPA group (91%) 10 mg UPA group (92%) Placebo (19%) Amenorrhoea (PBAC <2) in 10 days 5 mg UPA group (73%) 10 mg UPA group (82%) Placebo (6%) Median total change in fibroid volume 5 mg UPA group (−21.2%) 10 mg UPA group (−12.3%) Placebo (+3%) Self-reported pain scores improved compared to placebo Adverse effects comparable to placebo	Treatment with ulipristal acetate for 13 weeks effectively controlled excessive bleeding due to uterine fibroids and reduced the size of the fibroids.
PEARL II	Randomised Double Blind Double Dummy Active Comparator Controlled Noninferiority	Women with symptomatic fibroids; Age: 18–50 years BMI: 18–40 kg/m^2^; PBAC score >100 (first 8 days of menstruation); At least one uterine fibroid >3 cm but <10 cm; Fibroid uterus <16 weeks; Eligible for surgery	Women were randomised in a ratio of 1:1:1 to 5 mg/day UPA + placebo* (*n* = 97), 10 mg/day UPA + placebo (*n* = 104), placebo + Leuprorelin 3.75 mg IM monthly (*n* = 101) Treatment duration: 12–13 weeks *Placebo in UPA group - IM saline injection	PBAC score <75 was achieved in 5 mg UPA group (90%) 10 mg UPA group (98%) Leuprorelin (89%); Amenorrhoea (PBAC <2) was achieved 5 mg UPA group (75%) - Median time 7 days 10 mg UPA group (89%) - Median time 5 days Leuprorelin (80%) - Median time 21 days Median total change in fibroid volume 5 mg UPA group (−36%) 10 mg UPA group (−42%) Leuprorelin (−53%) Hot flushes (moderate to severe) 5 mg UPA group (11%) 10 mg UPA group (10%) Leuprorelin (40%) All three groups had improvements in pain and QoL scores	Treatment with ulipristal acetate for 13 weeks was noninferior to leuprolide acetate in controlling uterine bleeding and was associated with significantly lower risk of hot flushes.
PEARL III + Extension 1	PEARL III- Open label Extension 1 - Randomised double blind	Women with symptomatic fibroids; Age: 18–48 years; BMI: 18–40 kg/m^2^; Regular menstrual cycle; PBAC score >100 (first 8 days of menstruation); At least one uterine fibroid >3 cm but <10 cm; (FSH) <20 IU/L; Fibroid uterus <16 weeks; Eligible for surgery	PEARL III - UPA 10mg/day commenced in the first week of menstruation. Treatment duration 12 weeks (*n* = 209); Extension 1 - Up to 4 courses of 12 weeks of UPA treatment with drug free intervals, followed by randomised double blind treatment (1:1) to norethisterone acetate (NETA) or placebo for 10 days at the end of each treatment course. (*n* = 132)* *Participants recruited from the core PEARL III study	Amenorrhoea (PBAC <2) was achieved 1st UPA course (79.5%) - Median time 3.5 days 2nd UPA course (88.5%) - Median time 2 days 3rd UPA course (88.2%) - Median time 3 days 4th UPA course (89.7%) - Median time 3 days Median total change in fibroid volume 1st UPA course (−45.1%), *n* = 132 2nd UPA course (−63.2%), *n* = 131 3rd UPA course (−67%), *n* = 119 4th UPA course (−72.1%), *n* = 107 Hot flushes (moderate to severe) PAEC (6 weeks post treatment) 26% after course 1 25% after course 4 No effect of NETA on PAEC Improvement in pain scores appeared by the fifth week and was maintained during the four cycles. Quality-of-life scores were considerably reduced at the end of the treatment compared with scores at baseline and maintained throughout and at 3 months after cessation of treatment.	The study and its extensions (see subsequent section) demonstrates the safety and efficacy of repeated intermittent treatment of symptomatic fibroids with UPA.
PEARL III + Extension 2	PEARL III- Open label	Women with symptomatic fibroids; Age: 18–48 years; BMI: 18–40 kg/m^2^; Regular menstrual cycle; PBAC score >100 (first 8 days of menstruation); At least one uterine fibroid >3 cm but <10 cm; Fibroid uterus <16 weeks; Eligible for surgery; Completed PEARL III core study and extension 1	Extension 2 - Up to four additional courses of 12 weeks of UPA treatment with drug free intervals - TOTAL up to 8 courses (*n* = 64) * *Participants recruited from the core PEARL III study + completed extension 1	PAEC (assessed post treatment) 21% after course 4 16% after course 8 No changes in laboratory results outside normal ranges at any time	The study and its extensions demonstrate the safety and efficacy of repeated intermittent treatment of symptomatic fibroids with UPA.
PEARL IV	Randomised Double Blind Parallel-Group	Women with symptomatic fibroids; Age: 18–50 years; BMI: 18–40 kg/m^2^; Regular menstrual cycle; PBAC score >100 (first 8 days of menstruation); At least one uterine fibroid ≥3 cm but ≤12 cm; FSH <20 IU/L; Fibroid uterus <16 weeks	Women were randomised in a ratio of 1:1 to 5 mg/day UPA (*n* = 230) 10 mg/day UPA (*n* = 221) Treatment duration - Up to four courses of 12 weeks each (84 days) with a drug free interval between courses; until the start of the second menstrual bleed after course completion Women were followed up at 3 months after the fourth treatment course	Amenorrhoea (PBAC <2) was achieved 5 mg UPA group Course 1 - 71.8% Course 2 - 74.1% Course 3 - 73.3% Course 4 - 69.6% All four courses combined - 48.7% 10 mg UPA group Course 1- 82.6% Course 2 - 82.2% Course 3 - 78.3% Course 4 - 74.5% All 4 courses combined - 60.5% Median change in volume of the 3 largest fibroids at follow up 5 mg UPA group (−65%) 10 mg UPA group (−67.4%) Placebo (−53%) PAEC 5 mg UPA group Baseline - 7.8% Course 2 (16.3%) & Course 4 (16.2%) 3 months post UPA - 9.0% 10 mg UPA group Baseline - 8.4% Course 2 (19.2%) & Course 4 (10.3%) 3 months post UPA - 6.3% Both groups had improvements in pain and QoL scores Headaches and hot flushes were the most commonly reported side effects and occurred in ≤11% of patients, with the frequency of these events decreasing with each successive treatment course.	The study demonstrates the safety and efficacy of repeated intermittent treatment of symptomatic fibroids with UPA.
VENUS II	Randomised Double Blind Placebo Controlled Partial Crossover	Women with symptomatic fibroids; Age: 18–50 years; Regular menstrual cycle; MBL ≥80 mL measured using the alkali hematin method (first 8 days of menstruation) Minimum one discrete leiomyoma seen by TVUS FS) <20 IU/L Fibroid uterus ≤20 weeks	Women were randomised (*n* = 432) 5 mg/day UPA 10 mg/day UPA Placebo Randomized to one of six treatment arms in a 1:1:2:1:2:1 ratio, with course 1, course 2 dosing of placebo, ulipristal 5 mg; placebo, ulipristal 10 mg; ulipristal 5 mg, 5 mg; ulipristal 5 mg, placebo; ulipristal 10 mg, 10 mg; ulipristal 10 mg, placebo Treatment duration - Two courses of 3 months each. There was a two menses drug-free interval in between courses. Women were followed up at 3 months after treatment completion	Amenorrhoea was achieved 5 mg UPA group Course 1 - 42% Course 2 - 40.5% 10 mg UPA group Course 1- 54.8% Course 2 - 57.3% Placebo Course 1- 0% Course 2 - 8% Improvement from baseline in UFS-QOL revised activities subscale: 5-mg UPA: 48% 10-mg UPA: 57% Placebo: 13%	Consistent with VENUS I and the European studies, both doses of UPA were superior to placebo in the proportion of women achieving amenorrhea and time to amenorrhea.

The PEARL trials and VENUS trials differed in that; the PEARL trials were conducted in European Centres with a subset of participants that was predominantly Caucasian and had a strict BMI cut-off. The VENUS trials were conducted in the United States with a predominantly (70%) African American population of participants and no BMI cut-offs. As with the PEARL trials, VENUS trials also supported the meaningful positive impact of UPA on women’s quality of life (Lukes *et al*. 2019).

Real-world data are also available from an observational study – the A Prospective Multicenter Non-interventional Study of Women Treated With ESMYA (ulipristal acetate) as Preoperative Treatment of Moderate to Severe Symptoms of Uterine Fibroids (PREMYA) study involving 1473 women. Participants in this study received a 3-month course of 5 mg of UPA preoperatively. Only 38.8% of patients underwent surgery, mostly of a conservative/minimally invasive nature, and there were clinically relevant improvements in pain and health-related quality of life (HRQoL) scores (Fernandez *et al*. 2017).

## UPA and endometrial changes

**Figure 3:**
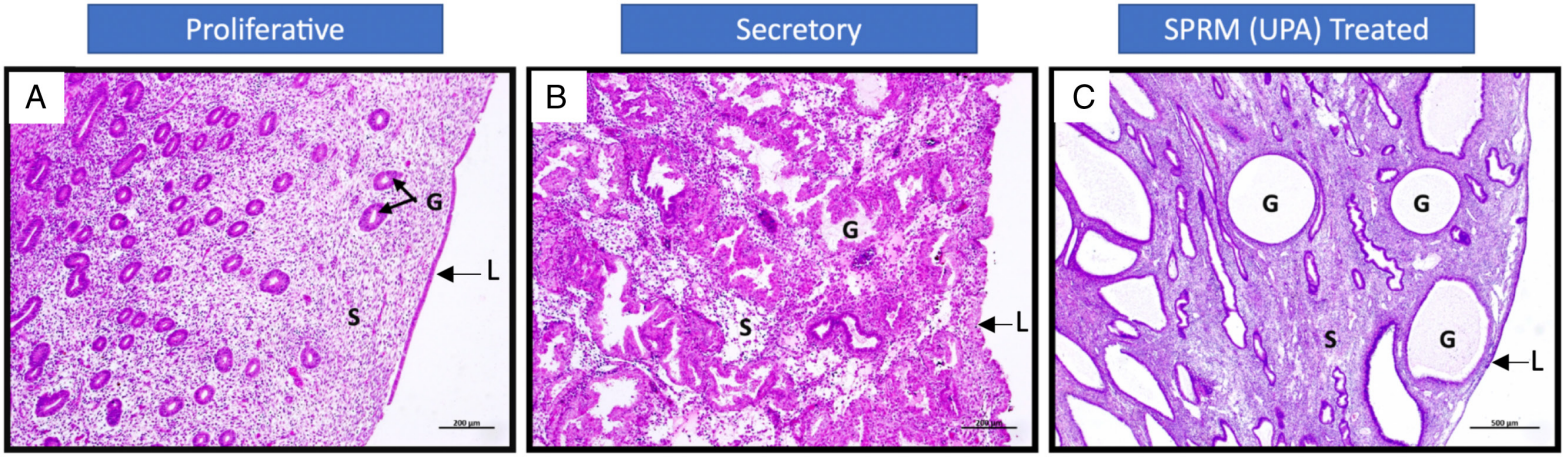
Haematoxylin and Eosin staining of the human endometrium in the proliferative phase (3A), secretory phase (3B) and following SPRM (UPA) treatment (3C) G: Endometrial Glands; S: Endometrial Stroma; L: Luminal Epithelium.

SPRMs have a progesterone antagonist effect and when used clinically there is a theoretical risk of unopposed endometrial oestrogen exposure and subsequent endometrial hyperplasia or cancer due to progesterone antagonism with use of SPRMs. Progesterone Receptor Modulator Associated Endometrial Changes (PAEC) are a spectrum of morphological endometrial effects seen with SPRM use, that is, representing a class effect with the use of the drugs. PAEC histology characteristically shows cystically dilated glands with non-physiological secretory appearances, inactive epithelium and few mitotic figures, in a background of a compact non-decidualised stroma (Williams *et al*. 2012) (Fig. 3).

UPA administration has been shown to affect the expression and localisation of endometrial sex steroid receptors, modulate progesterone-responsive genes and to reduce endometrial cell proliferation (Whitaker *et al*. 2017). There is limited information available on the impact of SPRMs, such as UPA on the human endometrium at the molecular/cellular level.

A recent systematic review has examined the endometrial effects of UPA use in ten studies involving 1450 women. The review supports the current understanding of PAEC; that it is essentially a benign condition, and reversible on discontinuation of UPA use. Most studies, however, have a limited follow-up period and have used UPA in up to four intermittent courses, so further research is needed before assuming that SPRMs including UPA are safe for long-term use (De Milliano *et al*. 2017).

## UPA and fibroids (morphological changes)

UPA is known to have proapoptotic, antifibrotic and antiproliferative effects on uterine fibroids (Xu *et al*. 2005, Courtoy *et al*. 2015, Donnez *et al*. 2018*b*). This is significant clinically, as fibroids treated with UPA that have reduced in size do not increase in size immediately after discontinuation of UPA use, unlike after treatment with GnRH agonists, where fibroid growth may recommence as early as 4 weeks following cessation of treatment (Donnez *et al*. 2012*b*).

UPA also alters the expression of angiogenic proteins, such as vascular endothelial growth factor, and reduces the amount of extracellular matrix by increasing matrix metalloproteinase (MMP) expression (Xu *et al*. 2008, Cox *et al*. 2018).

## UPA and surgery

There is good quality evidence to support the use of GnRH agonists preoperatively in women with uterine fibroids. The potential advantages include the reduction in fibroid and/or uterine size or volume, improvement in intraoperative blood loss, correction of pre-existing anaemia and the possibility of using more conservative approaches (e.g. vaginal hysterectomy) rather than a midline incision (Lethaby *et al*. 2002). The major disadvantage of using GnRH analogues for 3–4 months preoperatively is the risk of side effects – predominantly hot flushes and night sweats – due to oestrogen withdrawal. UPA has been compared to GnRH agonists for this purpose and found to have similar efficacy in controlling uterine bleeding and having reduced side effects (10% vs 40%) (Donnez *et al*. 2012*b*).

UPA is known to have proapoptotic, antifibrotic and antiproliferative effects on uterine fibroids with myometrial sparing (Gaillard *et al*. 1985, Xu *et al*. 2005, Courtoy *et al*. 2015, Donnez *et al*. 2018*b*). UPA is thought to also effect the fibroid pseudocapsule, which in turn is proposed to make surgical planes challenging to identify and subsequently result in difficult fibroid enucleation (Mallick *et al*. 2019). The pseudocapsule is a fibro-neurovascular structure surrounding the fibroid and separating it from the surrounding myometrium. In performing an ‘intracapsular myomectomy’, the fibroid is dissected from its pseudocapsule by breaking the connective tissue (fibrous) bridges (Tinelli *et al*. 2012*b*). An intracapsular myomectomy is recommended as it may subsequently reduce the risk of recurrence, uterine rupture and adhesion formation (Tinelli *et al*. 2012*a*). The distortion of the pseudocapsule with UPA use in some cases may increase the difficulty in correctly identifying the surgical planes between the fibroid and its surrounding pseudocapsule, making an intracapsular myomectomy challenging to perform.

Several clinical trials have evaluated the role of UPA prior to myomectomy and have differing outcomes. The MYOMEX trial (ulipristal acetate vs gonadotropin‐releasing hormone agonists prior to laparoscopic myomectomy) is a small randomised controlled trial (RCT) (*n* = 55) that compared GnRH analogues (leuprolide acetate 11.25 mg i.m. single dose + oral placebo tablet OD for 12 weeks) vs UPA (5 mg OD for 12 weeks + i.m. saline placebo injection) prior to a laparoscopic myomectomy. Women treated with UPA had higher intraoperative blood loss, longer suturing times for the first fibroid and the myomectomies were perceived to be subjectively more difficult (de Milliano *et al*. 2020). The MYOMEX trial was underpowered and included a very small number of women. Larger well-designed RCTs are needed before conclusions regarding the use of UPA prior to myomectomy procedures may be drawn. Another recent small (*n* = 10 UPA; *n* = 52 no pre-treatment) retrospective study in the United Kingdom supported the findings of potentially difficult laparoscopic myomectomy with UPA use (Mallick *et al*. 2019).

However, a recent systematic review which did not include the previously mentioned studies concluded that UPA is a suitable pre-treatment prior to both, hysteroscopic and laparoscopic myomectomies (Ferrero *et al*. 2019). The results must be interpreted with caution as the studies included are predominantly retrospective or prospective observational studies.

## UPA and endometriosis and adenomyosis

At the time of writing this review, we did not identify any RCTs evaluating the role of UPA in managing endometriosis and adenomyosis.

Endometriosis is a condition in which there is the presence of endometrial glands and stroma outside the uterus. It affects one in ten of women of reproductive age and is associated with pelvic pain, HMB and infertility (Eskenazi & Warner 1997, Missmer & Cramer 2003, Bedaiwy *et al*. 2009). There is conflicting and limited evidence regarding the role of UPA in endometriosis. In animal models (rats with surgically induced endometriosis), UPA was found to induce regression and atrophy of the endometriosis lesions. This was accompanied by upregulation of proapoptotic markers, reduced cell proliferation and inflammatory markers (Huniadi *et al*. 2013). A case report by Bressler *et al*. described a significant reduction in endometriosis related refractory chronic pelvic pain, when treated with high dose UPA for 3 months (Bressler *et al*. 2017). In contrast, Donnez *et al*. described excellent response to UPA treatment when administered for two 3-month courses with regards to reduction in fibroid size; however, no effect on an ovarian endometrioma, with both conditions co-existing in the same patient (Donnez & Dolmans 2016). The current understanding is that endometriosis lesions occur as superficial endometriosis, deep infiltrating endometriosis (DIE) and ovarian endometriosis (endometriomas). The aetiopathogenesis of these subtypes is poorly understood and some authors consider them as distinct clinical and pathological entities. There is conflicting evidence on the response of endometriosis to UPA with no clear demarcation between the three subtypes, and therefore, further investigation in the form of well-designed RCTs is necessary.

Bird *et al*. defined adenomyosis as ‘the benign invasion of the endometrium into the myometrium, producing a diffusely enlarged uterus which microscopically exhibits ectopic, non-neoplastic, endometrial glands and stroma surrounded by the hypertrophic and hyperplastic myometrium' (Bird *et al*. 1972). The prevalence of adenomyosis is difficult to ascertain because of a wide variation in diagnostic criteria both with imaging modalities and with histology. It has been estimated that histological confirmation of adenomyosis ranges from 5 to 70% of patients who undergo hysterectomy (Abbott 2017). With improvements in imaging technology, more cases of adenomyosis are now being diagnosed non-invasively both using 2D and 3D pelvic ultrasonography and MRI (Bluhm & Dueholm 2019, Liu *et al*. 2019).

There is an emerging concept of ‘progesterone resistance’ in the pathogenesis of these hormone-dependent conditions. In a normal cycling human endometrium, levels of the progesterone receptor (PR-A, PR-B; 2 isoforms) increase under the influence of the oestrogen exposure in the follicular phase of the cycle and the levels of the oestrogen receptor (ER) also increase. After ovulation, the levels of ER decline under the influence of rising circulating progesterone concentrations. In women with endometriosis, reduced endometrial PR-A expression compared to eutopic endometrium and an absence of PR-B were reported (Attia *et al*. 2000). This is likely a contributing mechanism, whereby progesterone does not trigger the expression of the endometrial steroid metabolising enzyme, 17 β hydroxysteroid dehydrogenase type 2 and subsequent metabolism of oestradiol (E2 - potent) to oestrone (E1 - less potent) (Bulun 2009, Bulun *et al*. 2010, Reis *et al*. 2013). Conversion of potent E2 to less potent E1, which normally occurs in the secretory phase endometrium, is regarded as a critical protective mechanism against oestrogen-induced growth. Moreover, endometrial expression profiling has documented dysregulation of progesterone-responsive genes in women with endometriosis (Taylor *et al*. 1999, Aghajanova *et al*. 2010).

Polycystic ovary syndrome (PCOS) is the most common endocrinopathy affecting women of reproductive age. Women with PCOS present with diverse features which includes those involving the reproductive system, such as, irregular menstrual cycles, hirsutism, infertility and pregnancy complications, along with metabolic features (insulin resistance (IR), metabolic syndrome, prediabetes, type 2 diabetes (DM2) and cardiovascular risk factors (Monash University 2018). Although the concept of altered response to endogenous progesterone (P4), ‘progesterone resistance’ has been addressed in the context of endometriosis, and it may be also evident in women with PCOS. A gene microanalysis by Savaris *et al*. reported that progesterone-regulated genes, including mitogen-inducible gene 6 (MIG6), leukemia inhibitory factor (LIF), GRB2-associated binding protein 1 (GAB1), S100P and claudin-4, were significantly lower in the endometrium of women with PCOS, whereas cell proliferation genes, such as Anillin and cyclin B1, were up-regulated. These data lend support to the concept of progesterone resistance (Savaris *et al*. 2011). The altered expression of the isoforms of the progesterone receptor (PR-A, PR-B) and the downstream signalling pathways has also been proposed as a mechanism for progesterone resistance in women with PCOS; however, further discussion is beyond the scope of this review (Li *et al*. 2014).

## Mifepristone (RU-486)

The first SPRM, RU486 (mifepristone), was discovered in the 1980s, during the quest for discovery for anti-glucocorticoid drugs (Moguilewsky & Philibert 1984) by the French pharmaceutical company Roussel-Uclaf. Mifepristone was synthesised by Georges Teutsch and is also referred as to RU-486, that is, RU-38486, the 38,486th compound synthesised by Roussel-Uclaf from 1949 to 1980, which has been shortened to RU-486. The drug was subsequently trialled for pregnancy interruption after its antiprogestin properties were investigated by Étienne-Émile Baulieu, a French endocrinologist and biochemist. Baulieu is often referred to as the ‘father’ of the abortion pill (Baulieu & Rosenblum 1991).

**Figure 4:**
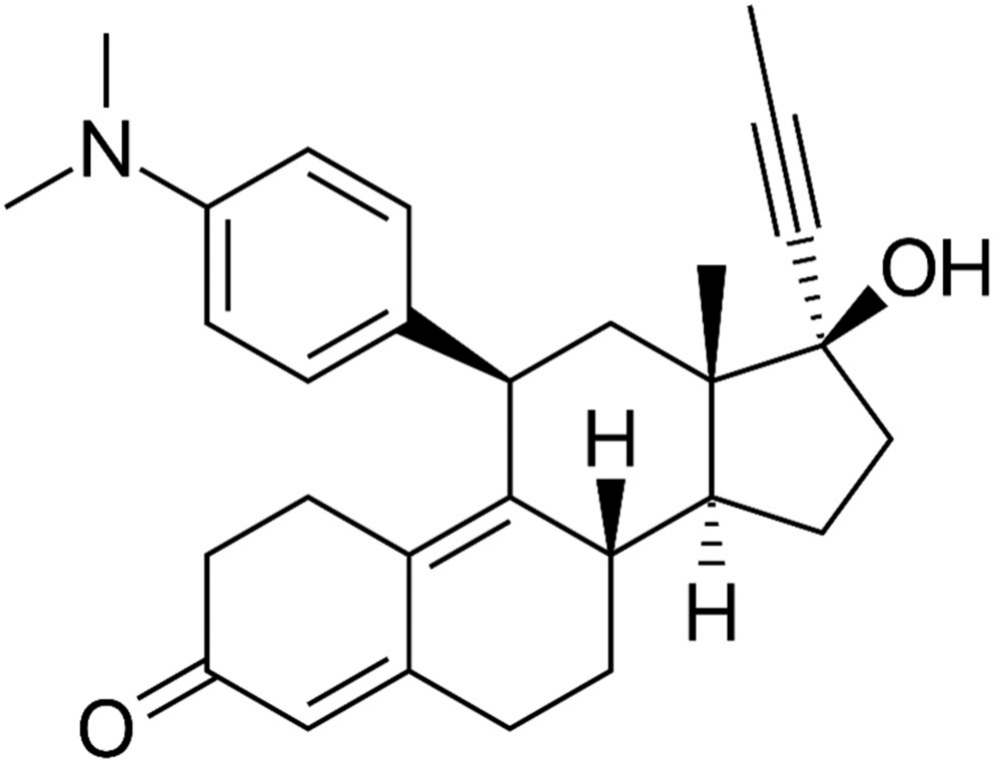
Chemical structure of mifepristone.

The chemical structure of mifepristone is shown in Fig. 4. It is a synthetic estrane steroid and its chemical name is 11β-(4-(dimethylamino) phenyl)-17α-(1-propynyl) estra-4,9-dien-17β-ol-3-one.

**Figure 5:**
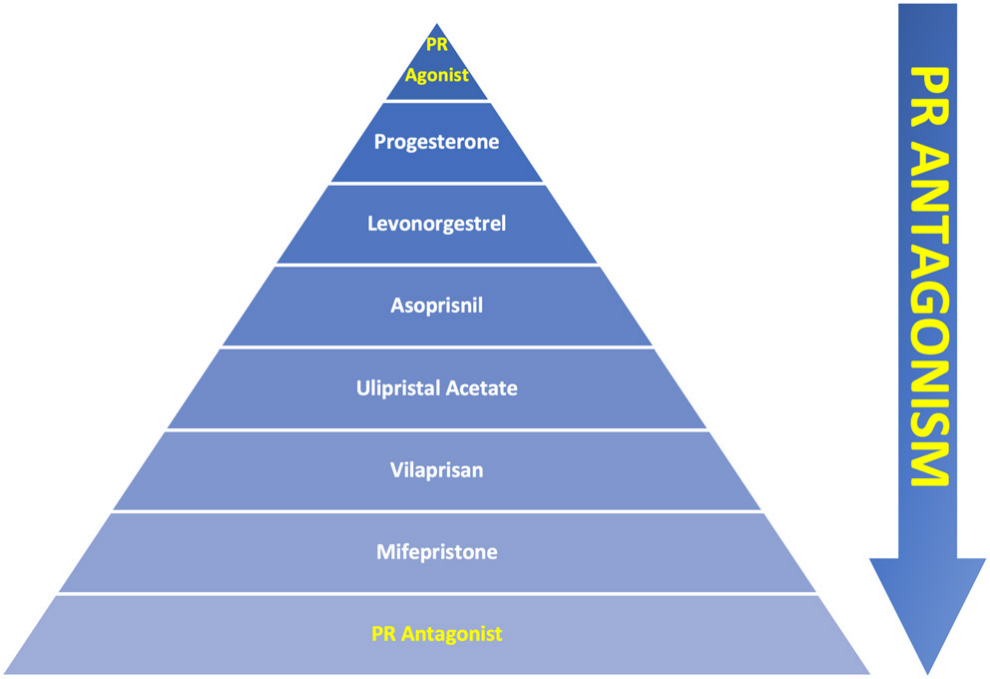
Spectrum of transition of progestational agonist to antagonist activity of SPRMS (PR: Progesterone Receptor). Progesterone is a pure PR agonist. Mifepristone is often classed (incorrectly) as a pure PR antagonist. The arrow demonstrates increasing levels of PR antagonism of the various SPRMs.

SPRMs may have mixed progestational agonist and antagonist activity, which can be assessed by the McPhail test conducted in immature female rabbits. The test consists of administration of oestradiol benzoate (day 1 to 6), followed by administration of the study drug (e.g. SPRM; day 7–12). Controls receive either the vehicle or oestradiol benzoate only. The rabbits are killed on day 15, and the mid portions of the uteri are then analysed histologically to assess changes in the endometrium, which are subsequently scored. Using this test, mifepristone is classed as a ‘pure’ antagonist (McPhail 1934, Elger *et al*. 2000, Chwalisz *et al*. 2005); however, it must be noted that in the absence of progesterone, mifepristone has a partial agonist effect. The test is now rarely used (Fig. 5).

In addition to its effects on PR, mifepristone is also a potent anti-glucocorticoid and a weak antiandrogen. It has a significantly higher affinity to the glucocorticoid receptor (GR) as compared to the endogenous corticosteroid cortisol (ten times) or dexamethasone (three times) (Philibert *et al*. 1985, Baulieu 2013). The anti-glucocorticoid actions are both centrally (ACTH feedback loop) and peripherally (via GR) mediated at doses >400 mg/day, administered as a single dose (Bertagna *et al*. 1984, Gaillard *et al*. 1985). Mifepristone is reported to be devoid of oestrogenic, anti-oestrogenic, mineralocorticoid and anti-mineralocorticoid properties.

## Mifepristone and pregnancy interruption

Mifepristone is the only SPRM that can interrupt pregnancy in several species, including humans, and is licensed for this purpose in several countries. The combination of mifepristone (usually 200 mg) and misoprostol, a prostaglandin analogue, is widely used for medical abortion in the first trimester (Cameron *et al*. 1986, Rodger *et al*. 1989, Raymond *et al*. 2013). Most focus has been on offering medical abortions to women ≤63 days of gestation, as these may be undertaken at home with great efficacy, safety and acceptability to women. This may be offered using a combination of 200 mg of oral mifepristone and vaginal or buccal misoprostol (Schaff *et al*. 2000, Chen & Creinin 2015).

More recent evidence also supports the role of using mifepristone-misoprostol for medical abortion from 64 to 70 days with a low rate of serious adverse events and high efficacy, also suitable in a ‘home setting’ (Hsia *et al*. 2019).

In the United Kingdom, the Royal College of Obstetricians and Gynaecologists (RCOG) suggests that medical abortion regimens using 200 mg oral mifepristone and misoprostol are effective and appropriate at any gestation, although as gestation increases, these would have to be undertaken in a medical facility (Royal College of Obstetricians and Gynaecologists 2011, National Institute for Health and Care Excellence 2019).

## Mifepristone and fibroids

The first clinical human trial (*n* = 10) to determine the effects of mifepristone in women with fibroids was published in 1993 and revealed that 50 mg/day administered over a 3-month period could shrink fibroids and was well tolerated (Murphy *et al*. 1993). The same team subsequently demonstrated that 25 mg/day dosing was optimal for this purpose and induced ovarian acyclicity (Murphy *et al*. 1995).

Since then, several studies have been published demonstrating the benefits of using mifepristone in women with fibroids. A recent meta-analysis (11 RCTs and 780 women) concluded that mifepristone significantly reduces uterine and fibroid (leiomyoma) volume and improves associated symptoms (HMB, dysmenorrhoea, pelvic pain, pressure and anaemia). The authors recommend 2.5 mg/day for 3 or 6 months as the optimum clinical treatment for uterine fibroids (leiomyoma). There is insufficient evidence to link its use to endometrial hyperplasia; however, monitoring of endometrial health should be undertaken (Shen *et al*. 2013).

In contrast, Cochrane (three RCTs and 112 women) concluded that mifepristone reduced HMB and improved fibroid-specific quality of life. However, it was not found to reduce fibroid volume and was associated with an increased risk of abnormalities in endometrial histology (Tristan *et al*. 2012). The endometrial changes are indistinguishable from PAEC induced by other SPRMs (Fiscella *et al*. 2011). 

## Mifepristone and Emergency Contraception

WHO defines ‘Emergency Contraception’ as methods of contraception that may be used to prevent pregnancy after sexual intercourse. The mechanism of action of mifepristone, however, depends upon its timing of administration in the menstrual cycle. When administered in the follicular phase, it prevents the LH surge and interrupts/delays ovulation (Spitz *et al*. 1996). If administered immediately at or after ovulation, it may block tubal motility and or blastocyst attachment or nidation as seen *in vitro*, that is, administration is contragestive rather than contraceptive (Lalitkumar *et al*. 2007).

Evidence suggests that mifepristone in a dose of 10 mg used up to 120 h after UPSI is an effective emergency contraceptive (Piaggio *et al*. 2003*a*). A Cochrane review has also supported the use of mifepristone as an emergency contraceptive (Cheng *et al*. 2008).

Higher doses are associated with a delay in menstruation (with added stress and anxiety of potential pregnancy), vaginal bleeds within 5 days of oral mifepristone and fatigue, and hence, the preference for the use of lower doses (Task Force on Postovulatory Methods of Fertility Regulation 1999).

A meta analysis has evaluated the administration of mifepristone at doses between 5 mg and 600 mg for the purpose of emergency contraception. The pregnancy rate increases by a factor of 1.6 when the dose of 10 mg is used instead of 25 mg. In terms of the number of women needed to treat, however, using 10 mg in the place of 25 mg implies having one extra pregnancy every 146 women requesting emergency contraception (Piaggio *et al*. 2003*b*).

## Mifepristone and long-term contraception

The first human clinical trial (*n* = 21) using mifepristone as a long-term contraceptive utilised a 200 mg single dose 48 h after an LH surge, determined by using urinary LH kits, with a treatment duration between 1 and 12 months. The endometrial effects were considered adequate to prevent pregnancy. The only major side effect was vaginal bleeding noted in a third of women, 2–3 days after treatment (Gemzell-Danielsson *et al*. 1993).

Evidence also shows that mifepristone at doses 2–5 mg/day (up to 120 days) has the potential to suppress the LH surge and subsequently ovulation, providing contraception and inducing amenorrhoea (Brown *et al*. 2002). A small study compared the daily administration of mifepristone 5 mg/day (*n* = 73) vs levonorgestrel 30 µg/day (progesterone only pill; *n* = 23) for 24 weeks. Mifepristone was found to be an effective contraceptive with improved bleeding patterns; however, benign endometrial changes (now described as PAEC) were noted with 6 months of use (Lakha *et al*. 2007). Mifepristone has also been evaluated as a ‘once-monthly’ pill; however, the results as a reliable contraceptive have been disappointing (Narvekar *et al*. 2006).

## Mifepristone and endometriosis

The first human clinical studies using the drug for women diagnosed with endometriosis showed promising results. The drug was used in a dose of 100 mg/day for 3 months (Kettel *et al*. 1991) or 50 mg/day for 6 months (Kettel *et al*. 1996). In the higher dose, short-term use group, improvement in endometriosis symptoms were noted, but no regression of endometriotic lesions was seen at post-treatment laparoscopy. In addition, evidence of hypercortisolism was noted. In the subsequent study, using a lower dose for a long duration, in addition to symptomatic improvement and regression of endometriotic lesions, a clear dissociation from the anti-glucocorticoid activity was noted.

Cochrane currently suggests that mifepristone improves endometriosis-associated dysmenorrhoea and potentially dyspareunia. Amenorrhoea is a common association and is classed as a ‘side effect’, although, lack of menstruation may be clearly beneficial in women with endometriosis-associated HMB. Doses <2.5 mg/day are less likely to be effective; however, based on the available evidence, clear conclusions on dosage cannot be made (Fu *et al*. 2017).

## Mifepristone and use for non-gynaecological indications

Although a full review of the non-gynaecological benefits of mifepristone is beyond the scope of this article, it has been explored and used in the clinical context for the indications discussed in the subsequent section.

Mifepristone has been explored as an anti-glucocorticoid drug. This may particularly be of value in the medical treatment of Cushing’s disease; mifepristone is considered as an adjuvant drug in this regard (Carmichael & Fleseriu 2013). The drug also shows potential for treating neuropsychiatric disorders, mood disorders and Alzheimer’s disease (DeBattista & Belanoff 2006). Mifepristone has also been trialled in the management of inoperable meningiomas (Haak *et al*. 1990, Matsuda *et al*. 1994).

For its antiprogesterone properties, mifepristone has been evaluated in the management of breast cancers (Romieu *et al*. 1987, Klijn *et al*. 1989). Treatment with RU486 (mifepristone) has been shown to prevent mammary tumorigenesis in *Brca1*/*Trp53*-deficient mice (Poole *et al*. 2006). The potential role of SPRMs in the prevention of breast cancer has been previously proposed (Bouchard *et al*. 2011).

## Vilaprisan

**Figure 6:**
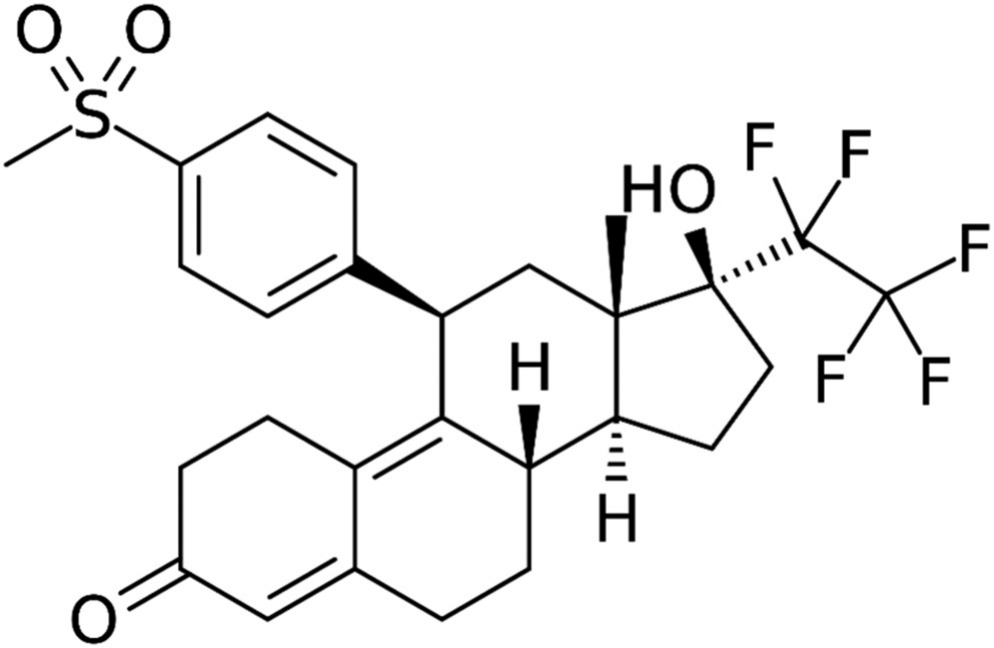
Chemical structure of vilaprisan.

Vilaprisan (BAY 1002670) is a more recent, potent, orally active SPRM. Vilaprisan was developed by and is the property of Bayer AG, Berlin, Germany. It is a 17-hydroxy-17-pentafluoroethyl-estra-4,9(10)- dien-11-aryl derivative. Vilaprisan can weakly bind to the glucocorticoid receptor and androgen receptor with no effect on the oestrogen receptor (Wagenfeld *et al*. 2013, Möller *et al*. 2018). Its chemical structure is shown in Fig. 6.

## Clinical trials

The currently available information for vilaprisan is generated from human clinical phase I and II trials in women with HMB and fibroids (Bradley *et al*. 2016, Schütt *et al*. 2016, 2018, Schultze-Mosgau *et al*. 2017, 2018). The results of the first trials comparing vilaprisan vs ulipristal acetate vs placebo in a randomised, double-blind, parallel-group fashion (ASTEROID 2 study) have not been published at the time of writing this paper. The findings from these trials are listed in Table 3.

**Table 3:** Summary of key findings of phase I and II clinical trials utilising vilaprisan.

Phase I clinical trials (Schütt *et al*. 2016, 2018)
Vilaprisan 0.5–5 mg/day for 12 weeks
1. Maximal non-bleeding rates achieved at dose of 2 mg or higher per day.
2. Doses >0.5 mg/day are associated with a decrease in FSH and LH.
3. Follicular growth mid-follicular oestradiol levels maintained.
4. Ovulation inhibited on >80% of participants at doses ≥1 mg/day.
5. Return of menstruation in ≤52 days after discontinuation.
6. No serious adverse events. A non-dose dependent transient rise in liver transaminases seen during treatment with vilaprisan which returned to baseline.
7. PAEC present in 10% of women pre-treatment and with 100% frequency in women on 5 mg/day (dose dependant). At doses of 1 mg/day, PAEC was observed in 70–90% of women. Regression was noted in majority of women at first bleed post treatment with regression in all participants at 4–6 months.
Phase II clinical trial (Bradley *et al*. 2016)
Vilaprisan 0.5–4 mg/day for 12 weeks
1. Amenorrhea (MBL <2 ml/28 days by alkali hematin) seen in 87–92% of participants at doses of 1 mg/day or higher.
2. Median time of amenorrhoea – 3 days.
3. Dose dependant reduction in fibroid size; up to 40% at 4 mg dose.
4. Improvements in HRQoL.
5. No serious adverse events.
6. PAEC seen in up to 40% of women on completing treatment with vilaprisan. Complete regression of PAEC to baseline levels was observed during the follow up period (24 weeks).

Vilaprisan has been undergoing clinical trials for management of HMB with uterine fibroids (ASTEROID 5; NCT Identifier: 03240523, ASTEROID 6; NCT Identifier: 03194646 and ASTEROID 7; NCT Identifier: 03699176) and a phase 2B randomised placebo-controlled trial in managing women with symptomatic endometriosis (NCT Identifier: 03573336; VILLENDO Study). At the time of writing this article, all vilaprisan trials were on hold due to new safety findings in long-term toxicology study in rodents (Bayer 2018, Burger 2018).

## Conclusion

SPRMs have been in development since the 1980s, and yet a perfect SPRM does not exist. The class of compounds do have some common effects, including suppression of the LH surge, anovulation, amenorrhoea and benign endometrial changes or PAEC. Some SPRMs have clear indications and therapeutic benefits, for example, mifepristone for pregnancy interruption and ulipristal acetate for emergency contraception and management of fibroid related HMB.

There still appears to be a great void in what SPRMs could achieve in terms of their therapeutic potential. There are few well-conducted RCTs which examine the role of SPRMs in endometriosis, adenomyosis or hormonally mediated chronic pain syndromes. For AUB alone, which is common (affecting one in four women of reproductive age) and debilitating, the role of SPRMs yet remains to be determined. The UCON study (Ulipristal vs Coil for the Management of Heavy Menstrual Bleeding; EudraCT: 2014-003408-65) once completed may provide valuable insights as to the utility of SPRM (UPA) administration in women with and without fibroids. SPRMs may also offer the potential of long-term oestrogen free contraception with less unscheduled bleeding – the side effect occurring in 20% of users of progestin-only methods of contraception (Lethaby *et al*. 2015). Optimal routes for administration for contraceptive indications will also require evaluation.

The attractiveness of SPRMS lies in the fact that they may be orally administered but have the potential for local drug delivery, that is, via intrauterine or vaginal routes. They have the advantage of maintaining peripheral mid-follicular oestradiol levels, avoiding hypo-oestrogenic side effects. From the perspective of the clinician, this means a class of drugs which may help in the medical management of common gynaecological pathologies without the side-effect profile of current standard medical treatments. From the academic perspective, very little is known about the endometrial mechanisms underpinning the development of PAEC and the long-term implications on the endometrial molecular signature.

These are reasons to further pursue research, development and undertake well-conducted clinical trials involving SPRMs with the ultimate goal of improving women’s health and their quality of life.

## Declaration of interest

H O D C has clinical research support for laboratory consumables and staff from Bayer AG and provides consultancy advice (but with no personal remuneration) for Bayer AG, PregLem SA, Gedeon Richter, Vifor Pharma UK Ltd, AbbVie Inc. and Myovant Sciences GmbH. H O D C receives royalties from UpToDate for the article on abnormal uterine bleeding. R R C has received support from Bayer AG as a clinical research fellow.

## Funding

This work did not receive any specific grant from any funding agency in the public, commercial or not-for-profit sector.

## References

[bib1] Abbott JA 2017 Adenomyosis and abnormal uterine bleeding (AUB-A)-pathogenesis, diagnosis, and management. Best Practice and Research: Clinical Obstetrics and Gynaecology 40 68–81. (10.1016/j.bpobgyn.2016.09.006) 27810281

[bib2] Aghajanova L Velarde MC Giudice LC 2010 Altered gene expression profiling in endometrium: evidence for progesterone resistance. Seminars in Reproductive Medicine 28 51–58. (10.1055/s-0029-1242994) 20104428

[bib3] Attia GR Zeitoun K Edwards D Johns A Carr BR Bulun SE 2000 Progesterone receptor isoform A but not B is expressed in endometriosis. Journal of Clinical Endocrinology and Metabolism 85 2897–2902. (10.1210/jcem.85.8.6739) 10946900

[bib4] Baulieu E-E 2013 The Antiprogestin Steroid RU 486 and Human Fertility Control. Springer Science & Business Media.

[bib5] Baulieu E-E Rosenblum M 1991 The ‘abortion pill’ RU-486, A Woman’s Choice. Simon & Schuster.

[bib6] Bayer 2018 Bayer annual report [Online]. (available at: https://www.bayer.com/en/bayer-annual-report-2018.pdfx). Accessed.

[bib7] Bedaiwy MA Abdel-Aleem MA Miketa A Falcone T 2009 Endometriosis: a critical appraisal of the advances and the controversies of a challenging health problem. Minerva Ginecologica 61 285–298. 19745795

[bib8] Bertagna X Bertagna C Luton JP Husson JM Girard F 1984 The new steroid analog RU 486 inhibits glucocorticoid action in man. Journal of Clinical Endocrinology and Metabolism 59 25–28. (10.1210/jcem-59-1-25) 6327758

[bib9] Bird CC Mcelin TW Manalo-Estrella P 1972 The elusive adenomyosis of the uterus – revisited. American Journal of Obstetrics and Gynecology 112 583–593. (10.1016/0002-9378(72)90781-8) 5059589

[bib10] Bluhm M Dueholm M 2019 Imaging for adenomyosis-Making the diagnosis by sonography. Journal of Minimally Invasive Gynecology 27 267 (10.1016/j.jmig.2019.10.001) 31610319

[bib11] Bouchard P 2014 Selective progesterone receptor modulators: a class with multiple actions and applications in reproductive endocrinology, and gynecology. Gynecological Endocrinology 30 683–684. (10.3109/09513590.2014.950647) 25242338

[bib12] Bouchard P Chabbert-Buffet N Fauser BC 2011 Selective progesterone receptor modulators in reproductive medicine: pharmacology, clinical efficacy and safety. Fertility and Sterility 96 1175–1189. (10.1016/j.fertnstert.2011.08.021) 21944187

[bib13] Bradley L Ren X Groettrup-Wolfers E Petersdorf K Seitz C 2016 Results of the asteroid (assess safety and efficacy of vilaprisan in patients with uterine fibroids) 1 study: a phase 2, placebo-controlled dose finding study. Fertility and Sterility 106 e95–e96. (10.1016/j.fertnstert.2016.07.278) 30527839

[bib14] Bressler LH Bernardi LA Snyder MA Wei JJ Bulun S 2017 Treatment of endometriosis-related chronic pelvic pain with ulipristal acetate and associated endometrial changes. HSOA Journal of Reproductive Medicine Gynaecology and Obstetrics 2 008 (10.24966/RMGO-2574/100008) 30680372 PMC6342495

[bib15] Brown A Cheng L Lin S Baird DT 2002 Daily low-dose mifepristone has contraceptive potential by suppressing ovulation and menstruation: a double-blind randomized control trial of 2 and 5 mg per day for 120 days. Journal of Clinical Endocrinology and Metabolism 87 63–70. (10.1210/jcem.87.1.8140) 11788624

[bib16] Bulun SE 2009 Endometriosis. New England Journal of Medicine 360 268–279. (10.1056/NEJMra0804690) 19144942

[bib17] Bulun SE Cheng YH Pavone ME Yin P Imir G Utsunomiya H Thung S Xue Q Marsh EE Tokunaga H, ***et al*** 2010 17β-Hydroxysteroid dehydrogenase-2 deficiency and progesterone resistance in endometriosis. Seminars in Reproductive Medicine 28 44–50. (10.1055/s-0029-1242992) 20108182 PMC4511594

[bib18] Burger L 2018 Bayer Halts Vilaprisan Drug Trials Due to Toxicology Data. Reuters.

[bib19] Cameron IT Michie AF Baird DT 1986 Therapeutic abortion in early pregnancy with antiprogestogen RU486 alone or in combination with prostaglandin analogue (gemeprost). Contraception 34 459–468. (10.1016/0010-7824(86)90055-7) 3816230

[bib20] Carmichael JD Fleseriu M 2013 Mifepristone: is there a place in the treatment of Cushing’s disease? Endocrine 44 20–32. (10.1007/s12020-012-9846-1) 23192246

[bib21] Catherino WH Malik M Driggers P Chappel S Segars J Davis J 2010 Novel, orally active selective progesterone receptor modulator CP8947 inhibits leiomyoma cell proliferation without adversely affecting endometrium or myometrium. Journal of Steroid Biochemistry and Molecular Biology 122 279–286. (10.1016/j.jsbmb.2010.05.005) 20493256 PMC3576019

[bib22] Chen MJ Creinin MD 2015 Mifepristone with buccal misoprostol for medical abortion: a systematic review. Obstetrics and Gynecology 126 12–21. (10.1097/AOG.0000000000000897) 26241251

[bib24] Cheng L Gulmezoglu AM Piaggio G Ezcurra E Van Look PF 2008 Interventions for emergency contraception. Cochrane Database of Systematic Reviews CD001324 (10.1002/14651858.CD001324.pub3) 15266446

[bib23] Cheng L Che Y Gulmezoglu AM 2012 Interventions for emergency contraception. Cochrane Database of Systematic Reviews CD001324 (10.1002/14651858.CD001324.pub4) 22895920

[bib25] Chwalisz K Perez MC Demanno D Winkel C Schubert G Elger W 2005 Selective progesterone receptor modulator development and use in the treatment of leiomyomata and endometriosis. Endocrine Reviews 26 423–438. (10.1210/er.2005-0001) 15857972

[bib26] Cleland K Zhu H Goldstuck N Cheng L Trussell J 2012 The efficacy of intrauterine devices for emergency contraception: a systematic review of 35 years of experience. Human Reproduction 27 1994–2000. (10.1093/humrep/des140) 22570193 PMC3619968

[bib27] Courtoy GE Donnez J Marbaix E Dolmans MM 2015 In vivo mechanisms of uterine myoma volume reduction with ulipristal acetate treatment. Fertility and Sterility 104 426.e1–434.e1. (10.1016/j.fertnstert.2015.04.025) 26003270

[bib28] Cox J Malik M Britten J Lewis T Catherino WH 2018 Ulipristal acetate and extracellular matrix production in human leiomyomas in vivo: a laboratory analysis of a randomized placebo controlled trial. Reproductive Sciences 25 198–206. (10.1177/1933719117728802) 28929861 PMC5933104

[bib30] De Milliano I van Hattum D Ket JCF Huirne JAF Hehenkamp WJK 2017 Endometrial changes during ulipristal acetate use: a systematic review. European Journal of Obstetrics, Gynecology, and Reproductive Biology 214 56–64. (10.1016/j.ejogrb.2017.04.042) 28482329

[bib29] de Milliano I Huirne JAF Thurkow AL Radder C Bongers MY Van Vliet H Van De Lande J van de Ven PM Hehenkamp WJK 2020 Ulipristal acetate vs gonadotropin-releasing hormone agonists prior to laparoscopic myomectomy (MYOMEX trial): short-term results of a double-blind randomized controlled trial. Acta Obstetricia and Gynecologica Scandinavica 99 89–98. (10.1111/aogs.13713) PMC6973004 31468503

[bib31] DeBattista C Belanoff J 2006 The use of mifepristone in the treatment of neuropsychiatric disorders. Trends in Endocrinology and Metabolism 17 117–121. (10.1016/j.tem.2006.02.006) 16530421

[bib32] Donnez J 2018 Liver injury and ulipristal acetate: an overstated tragedy? Fertility and Sterility 110 593–595. (10.1016/j.fertnstert.2018.06.044) 30196943

[bib35] Donnez J Dolmans MM 2016 Uterine fibroid management: from the present to the future. Human Reproduction Update 22 665–686. (10.1093/humupd/dmw023) 27466209 PMC5853598

[bib37] Donnez J Tatarchuk TF Bouchard P Puscasiu L Zakharenko NF Ivanova T Ugocsai G Mara M Jilla MP Bestel E, ***et al*** 2012a Ulipristal acetate versus placebo for fibroid treatment before surgery. New England Journal of Medicine 366 409–420. (10.1056/NEJMoa1103182) 22296075

[bib38] Donnez J Tomaszewski J Vazquez F Bouchard P Lemieszczuk B Baro F Nouri K Selvaggi L Sodowski K Bestel E, ***et al*** 2012b Ulipristal acetate versus leuprolide acetate for uterine fibroids. New England Journal of Medicine 366 421–432. (10.1056/NEJMoa1103180) 22296076

[bib39] Donnez J Vazquez F Tomaszewski J Nouri K Bouchard P Fauser BC Barlow DH Palacios S Donnez O Bestel E, ***et al*** 2014 Long-term treatment of uterine fibroids with ulipristal acetate. Fertility and Sterility 101 1565.e1–1573.e1. (10.1016/j.fertnstert.2014.02.008) 24630081

[bib36] Donnez J Hudecek R Donnez O Matule D Arhendt HJ Zatik J Kasilovskiene Z Dumitrascu MC Fernandez H Barlow DH, ***et al*** 2015 Efficacy and safety of repeated use of ulipristal acetate in uterine fibroids. Fertility and Sterility 103 519.e3–527.e3. (10.1016/j.fertnstert.2014.10.038) 25542821

[bib33] Donnez J Arriagada P Marciniak M Larrey D 2018a Liver safety parameters of ulipristal acetate for the treatment of uterine fibroids: a comprehensive review of the clinical development program. Expert Opinion on Drug Safety 17 1225–1232. (10.1080/14740338.2018.1550070) 30460871

[bib34] Donnez J Dolmans MM Courtoy GE 2018b Molecular mechanisms responsible for myoma volume reduction after ulipristal acetate. Fertility and Sterility 110 e140–e141. (10.1016/j.fertnstert.2018.07.415) 26003270

[bib40] Elger W Bartley J Schneider B Kaufmann G Schubert G Chwalisz K 2000 Endocrine pharmacological characterization of progesterone antagonists and progesterone receptor modulators with respect to PR-agonistic and antagonistic activity. Steroids 65 713–723. (10.1016/s0039-128x(00)00178-1) 11108882

[bib41] Eskenazi B Warner ML 1997 Epidemiology of endometriosis. Obstetrics and Gynecology Clinics of North America 24 235–258. (10.1016/s0889-8545(05)70302-8) 9163765

[bib42] European Consortium for Emergency Contraception 2017 Emergency contraception in the United Kingdom [Online]. (available at: http://www.ec-ec.org/emergency-contraception-in-europe/country-by-country-information-2/united-kingdom/#pub4). Accessed.

[bib43] European Medicines Agency 2018 Esmya: new measures to minimise risk of rare but serious liver injury [Online]. (available at: https://www.ema.europa.eu/documents/referral/esmya-article-20-procedure-esmya-new-measures-minimise-risk-rare-serious-liver-injury_en-0.pdf). Accessed.

[bib44] Fernandez H Schmidt T Powell M Costa AP Arriagada P Thaler C 2017 Real world data of 1473 patients treated with ulipristal acetate for uterine fibroids: Premya study results. European Journal of Obstetrics, Gynecology, and Reproductive Biology 208 91–96. (10.1016/j.ejogrb.2016.11.003) 27898340

[bib45] Ferrero S Vellone VG Barra F Scala C 2019 Ulipristal acetate before hysteroscopic and laparoscopic surgery for uterine myomas: help or hindrance? Gynecologic and Obstetric Investigation 84 313–325. (10.1159/000495347) 30554215

[bib46] Fiscella J Bonfiglio T Winters P Eisinger SH Fiscella K 2011 Distinguishing features of endometrial pathology after exposure to the progesterone receptor modulator mifepristone. Human Pathology 42 947–953. (10.1016/j.humpath.2010.11.003) 21315422 PMC3118265

[bib47] Frick KD Clark MA Steinwachs DM Langenberg P Stovall D Munro MG Dickersin K & Group S-DR 2009 Financial and quality-of-life burden of dysfunctional uterine bleeding among women agreeing to obtain surgical treatment. Women’s Health Issues 19 70–78. (10.1016/j.whi.2008.07.002) 19111789

[bib48] Fu J Song H Zhou M Zhu H Wang Y Chen H Huang W 2017 Progesterone receptor modulators for endometriosis. Cochrane Database of Systematic Reviews 7 CD009881 (10.1002/14651858.CD009881.pub2) 28742263 PMC6483151

[bib49] Gaillard RC Poffet D Riondel AM Saurat JH 1985 RU 486 inhibits peripheral effects of glucocorticoids in humans. Journal of Clinical Endocrinology and Metabolism 61 1009–1011. (10.1210/jcem-61-6-1009) 4055982

[bib50] Geary RS Gurol-Urganci I Kiran A Cromwell DA Bansi-Matharu L Shakespeare J Mahmood T van der Meulen J 2019 Factors associated with receiving surgical treatment for menorrhagia in England and Wales: findings from a cohort study of the National Heavy Menstrual Bleeding Audit. BMJ Open 9 e024260. 10.1136/bmjopen-2018-024260 PMC6377553 30782899

[bib51] Gemzell-Danielsson K Swahn ML Svalander P Bygdeman M 1993 Early luteal phase treatment with mifepristone (RU 486) for fertility regulation. Human Reproduction 8 870–873. (10.1093/oxfordjournals.humrep.a138157) 8345076

[bib52] Glasier A 1997 Emergency postcoital contraception. New England Journal of Medicine 337 1058–1064. (10.1056/NEJM199710093371507) 9321535

[bib53] Glasier AF Cameron ST Fine PM Logan SJ Casale W Van Horn J Sogor L Blithe DL Scherrer B Mathe H, ***et al*** 2010 Ulipristal acetate versus levonorgestrel for emergency contraception: a randomised non-inferiority trial and meta-analysis. Lancet 375 555–562. (10.1016/S0140-6736(10)60101-8) 20116841

[bib54] Guo SW Groothuis PG 2018 Is it time for a paradigm shift in drug research and development in endometriosis/adenomyosis? Human Reproduction Update 24 577–598. (10.1093/humupd/dmy020) 29893860

[bib55] Haak HR De Keizer RJ Hagenouw-Taal JC Van Seters AP Vielvoye GJ Van Dulken H 1990 Successful mifepristone treatment of recurrent, inoperable meningioma. Lancet 336 124–125. (10.1016/0140-6736(90)91647-s) 1975312

[bib56] Hallberg L Nilsson L 1964 Determination of menstrual blood loss. Scandinavian Journal of Clinical and Laboratory Investigation 16 244–248. (10.3109/00365516409060511) 14161862

[bib57] Hsia JK Lohr PA Taylor J Creinin MD 2019 Medical abortion with mifepristone and vaginal misoprostol between 64 and 70 days’ gestation. Contraception 100 178–181. (10.1016/j.contraception.2019.05.006) 31102629

[bib58] Huang Y Jensen JT Brache V Cochon L Williams A Miranda MJ Croxatto H Kumar N Sussman H Hoskin E, ***et al*** 2014 A randomized study on pharmacodynamic effects of vaginal rings delivering the progesterone receptor modulator ulipristal acetate: research for a novel estrogen-free, method of contraception. Contraception 90 565–574. (10.1016/j.contraception.2014.08.006) 25193534 PMC4253673

[bib59] Huniadi CA Pop OL Antal TA Stamatian F 2013 The effects of ulipristal on Bax/Bcl-2, cytochrome c, Ki-67 and cyclooxygenase-2 expression in a rat model with surgically induced endometriosis. European Journal of Obstetrics, Gynecology, and Reproductive Biology 169 360–365. (10.1016/j.ejogrb.2013.03.022) 23619346

[bib60] Illingworth BJG Hirsch M Duffy JMN 2018 Ulipristal acetate for treatment of symptomatic uterine leiomyomas: a randomized controlled trial. Obstetrics and Gynecology 132 215 (10.1097/AOG.0000000000002718) 29939915

[bib61] Jensen JT 2013 Vaginal ring delivery of selective progesterone receptor modulators for contraception. Contraception 87 314–318. (10.1016/j.contraception.2012.08.038) 23040126 PMC3657703

[bib62] Keenan JA 2011 Ulipristal acetate: contraceptive or contragestive? Annals of Pharmacotherapy 45 813–815. (10.1345/aph.1Q248) 21666088

[bib64] Kettel LM Murphy AA Mortola JF Liu JH Ulmann A Yen SS 1991 Endocrine responses to long-term administration of the antiprogesterone RU486 in patients with pelvic endometriosis. Fertility and Sterility 56 402–407. (10.1016/s0015-0282(16)54531-2) 1716596

[bib63] Kettel LM Murphy AA Morales AJ Ulmann A Baulieu EE Yen SS 1996 Treatment of endometriosis with the antiprogesterone mifepristone (RU486). Fertility and Sterility 65 23–28. (10.1016/s0015-0282(16)58022-4) 8557150

[bib65] Klijn JG De Jong FH Bakker GH Lamberts SW Rodenburg CJ Alexieva-Figusch J 1989 Antiprogestins, a new form of endocrine therapy for human breast cancer. Cancer Research 49 2851–2856. 2720645

[bib66] Lakha F Ho PC Van Der Spuy ZM Dada K Elton R Glasier AF Critchley HO Williams AR Baird DT 2007 A novel estrogen-free oral contraceptive pill for women: multicentre, double-blind, randomized controlled trial of mifepristone and progestogen-only pill (levonorgestrel). Human Reproduction 22 2428–2436. (10.1093/humrep/dem177) 17609247

[bib67] Lalitkumar PG Lalitkumar S Meng CX Stavreus-Evers A Hambiliki F Bentin-Ley U Gemzell-Danielsson K 2007 Mifepristone, but not levonorgestrel, inhibits human blastocyst attachment to an in vitro endometrial three-dimensional cell culture model. Human Reproduction 22 3031–3037. (10.1093/humrep/dem297) 17890724

[bib69] Lethaby A Vollenhoven B Sowter M 2002 Efficacy of pre-operative gonadotrophin hormone releasing analogues for women with uterine fibroids undergoing hysterectomy or myomectomy: a systematic review. BJOG 109 1097–1108. (10.1111/j.1471-0528.2002.01225.x) 12387461

[bib68] Lethaby A Hussain M Rishworth JR Rees MC 2015 Progesterone or progestogen-releasing intrauterine systems for heavy menstrual bleeding. Cochrane Database of Systematic Reviews CD002126 (10.1002/14651858.CD002126.pub3) 25924648

[bib70] Leung VW Soon JA Lynd LD Marra CA Levine M 2016 Population-based evaluation of the effectiveness of two regimens for emergency contraception. International Journal of Gynaecology and Obstetrics 133 342–346. (10.1016/j.ijgo.2015.10.017) 26969148

[bib71] Lewis TD Malik M Britten J San Pablo AM Catherino WH 2018 A comprehensive review of the pharmacologic management of uterine leiomyoma. BioMed Research International 2018 2414609 (10.1155/2018/2414609) 29780819 PMC5893007

[bib73] Li X Feng Y Lin JF Billig H Shao R 2014 Endometrial progesterone resistance and PCOS. Journal of Biomedical Science 21 2 (10.1186/1423-0127-21-2) 24405633 PMC3917599

[bib72] Li HWR Resche-Rigon M Bagchi IC Gemzell-Danielsson K Glasier A 2019 Does ulipristal acetate emergency contraception (ella®) interfere with implantation? Contraception 100 386–390. (10.1016/j.contraception.2019.07.140) 31351035

[bib74] Lira-Albarran S Durand M Barrera D Vega C Becerra RG Diaz L Garcia-Quiroz J Rangel C Larrea F 2018 A single preovulatory administration of ulipristal acetate affects the decidualization process of the human endometrium during the receptive period of the menstrual cycle. Molecular and Cellular Endocrinology 476 70–78. (10.1016/j.mce.2018.04.010) 29709683

[bib75] Liu L Wong L Mol B Condous G Costa F Leonardi M 2019 Diagnostic accuracy of transvaginal ultrasound and magnetic resonance imaging for the diagnosis of adenomyosis: systematic review and meta-analysis. Ultrasound in Medicine and Biology 45 (Supplement 1) S54 (10.1016/j.ultrasmedbio.2019.07.592) 33502767

[bib76] Lu Y Ji D Yao X Wei X Liang X 2015 CHEMDNER system with mixed conditional random fields and multi-scale word clustering. Journal of Cheminformatics 7 (Supplement 1) S4 (10.1186/1758-2946-7-S1-S4) 25810775 PMC4331694

[bib77] Lukes AS Soper D Harrington A Sniukiene V Mo Y Gillard P Shulman L 2019 Health-related quality of life with ulipristal acetate for treatment of uterine leiomyomas: a randomized controlled trial. Obstetrics and Gynecology 133 869–878. (10.1097/AOG.0000000000003211) 30969201 PMC6485305

[bib78] Lusher SJ Raaijmakers HC Vu-Pham D Dechering K Lam TW Brown AR Hamilton NM Nimz O Bosch R Mcguire R, ***et al*** 2011 Structural basis for agonism and antagonism for a set of chemically related progesterone receptor modulators. Journal of Biological Chemistry 286 35079–35086. (10.1074/jbc.M111.273029) 21849509 PMC3186393

[bib79] Mallick R Oxley S Odejinmi F 2019 The use of ulipristal acetate (Esmya) prior to laparoscopic myomectomy: help or hindrance? Gynecology and Minimally Invasive Therapy 8 62–66. (10.4103/GMIT.GMIT_79_18) 31143625 PMC6515756

[bib80] Matsuda Y Kawamoto K Kiya K Kurisu K Sugiyama K Uozumi T 1994 Antitumor effects of antiprogesterones on human meningioma cells in vitro and in vivo. Journal of Neurosurgery 80 527–534. (10.3171/jns.1994.80.3.0527) 8113866

[bib81] McEwan IJ 2009 Nuclear receptors: one big family. Methods in Molecular Biology 505 3–18. (10.1007/978-1-60327-575-0_1) 19117136

[bib82] McPhail MK 1934 The assay of progestin. Journal of Physiology 83 145–156. (10.1113/jphysiol.1934.sp003217) 16994619 PMC1394321

[bib83] Medicines and Healthcare Products Regulatory Agency 2018 Esmya (ulipristal acetate) for symptoms of uterine fibroids: restrictions to use and requirement to check liver function before, during and after treatment [Online]. (available at: https://www.gov.uk/drug-safety-update/esmya-ulipristal-acetate-and-risk-of-serious-liver-injury-new-restrictions-to-use-and-requirements-for-liver-function-monitoring-before-during-and-after-treatment). Accessed.

[bib84] Missmer SA Cramer DW 2003 The epidemiology of endometriosis. Obstetrics and Gynecology Clinics of North America 30 1–19, vii (10.1016/s0889-8545(02)00050-5) 12699255

[bib85] Moguilewsky M Philibert D 1984 RU 38486: potent antiglucocorticoid activity correlated with strong binding to the cytosolic glucocorticoid receptor followed by an impaired activation. Journal of Steroid Biochemistry 20 271–276. (10.1016/0022-4731(84)90216-4) 6708512

[bib86] Möller C Bone W Cleve A Klar U Rotgeri A Rottmann A Schultze-Mosgau MH Wagenfeld A Schwede W 2018 Discovery of Vilaprisan (BAY 1002670): a highly potent and selective progesterone receptor modulator optimized for gynecologic therapies. ChemMedChem 13 2271–2280. (10.1002/cmdc.201800487) 30407750 PMC6282584

[bib87] Monash University 2018 International evidence-based guideline for the assessment and management of polycystic ovary syndrome [Online]. (available at: https://www.monash.edu/__data/assets/pdf_file/0004/1412644/PCOS_Evidence-Based-Guidelines_20181009.pdf). Accessed.

[bib88] Murphy AA Kettel LM Morales AJ Roberts VJ Yen SS 1993 Regression of uterine leiomyomata in response to the antiprogesterone RU 486. Journal of Clinical Endocrinology and Metabolism 76 513–517. (10.1210/jcem.76.2.8432797) 8432797

[bib89] Murphy AA Morales AJ Kettel LM Yen SS 1995 Regression of uterine leiomyomata to the antiprogesterone RU486: dose-response effect. Fertility and Sterility 64 187–190. (10.1016/S0015-0282(16)57678-X) 7789557

[bib90] Nallasamy S Kim J Sitruk-Ware R Bagchi M Bagchi I 2013 Ulipristal blocks ovulation by inhibiting progesterone receptor-dependent pathways intrinsic to the ovary. Reproductive Sciences 20 371–381. (10.1177/1933719112459239) 23012316 PMC3676258

[bib91] Narvekar N Glasier A Dada K Van der Spuy Z Ho PC Cheng L Baird DT 2006 Toward developing a once-a-month pill: a double-blind, randomized, controlled trial of the effect of three single doses of mifepristone given at midcycle on the pattern of menstrual bleeding. Fertility and Sterility 86 819–824. (10.1016/j.fertnstert.2006.02.115) 17027354

[bib92] National Institute for Health and Care Excellence 2019 Abortion care (NG140) [Online]. (available at: https://www.nice.org.uk/guidance/ng140/resources/abortion-care-pdf-66141773098693). Accessed.

[bib93] NICE 2018 NG88: Heavy Menstrual Bleeding: Assessment and Management. National Institute for Health and Clinical Excellence (NICE). 29634173

[bib94] Office for National Statistics 2017 Birth characteristics in England and Wales: 2017 [Online]. (available at: https://www.ons.gov.uk/peoplepopulationandcommunity/birthsdeathsandmarriages/livebirths/bulletins/birthcharacteristicsinenglandandwales/2017/pdf). Accessed.

[bib95] Passaro M Piquion J Mullen N Sutherland D Alexander N Nieman L 1997 Safety and luteal phase effects of the antiprogestin CDB-2914 in normally cycling women. Endocrine Society Abstracts **227 **1–370.

[bib96] Passaro MD Piquion J Mullen N Sutherland D Zhai S Figg WD Blye R Nieman LK 2003 Luteal phase dose-response relationships of the antiprogestin CDB-2914 in normally cycling women. Human Reproduction 18 1820–1827. (10.1093/humrep/deg342) 12923133

[bib97] Philibert D Moguilewsky M Mary I Lecaque D Tournemine C Secchi J Deraedt R 1985 Pharmacological profile of RU 486 in animals. In The Antiprogestin Steroid RU 486 and Human Fertility Control. Springer.

[bib98] Piaggio G Heng Z Von Hertzen H Bilian X Linan C 2003a Combined estimates of effectiveness of mifepristone 10 mg in emergency contraception. Contraception 68 439–446. (10.1016/s0010-7824(03)00110-0) 14698074

[bib99] Piaggio G Von Hertzen H Heng Z Bilian X Cheng L 2003b Meta-analyses of randomized trials comparing different doses of mifepristone in emergency contraception. Contraception 68 447–452. (10.1016/s0010-7824(03)00142-2) 14698075

[bib100] Pincus G 1960 Fertility control by endocrine agents. European Journal of Endocrinology 34 S135–S138. 10.1530/acta.0.xxxivs135 14486711

[bib101] Poole AJ Li Y Kim Y Lin SC Lee WH Lee EY 2006 Prevention of BRCA1-mediated mammary tumorigenesis in mice by a progesterone antagonist. Science 314 1467–1470. (10.1126/science.1130471) 17138902

[bib102] Raymond EG Shannon C Weaver MA Winikoff B 2013 First-trimester medical abortion with mifepristone 200 mg and misoprostol: a systematic review. Contraception 87 26–37. (10.1016/j.contraception.2012.06.011) 22898359

[bib103] Reel JR Hild-Petito S Blye RP 1998 Antiovulatory and postcoital antifertility activity of the antiprogestin CDB-2914 when administered as single, multiple, or continuous doses to rats. Contraception 58 129–136. (10.1016/s0010-7824(98)00067-5) 9773268

[bib104] Reis FM Petraglia F Taylor RN 2013 Endometriosis: hormone regulation and clinical consequences of chemotaxis and apoptosis. Human Reproduction Update 19 406–418. (10.1093/humupd/dmt010) 23539633 PMC3682670

[bib105] Rodger MW Flogan AF Baird DT 1989 Induction of early abortion with mifepristone (RU486) and two different doses of prostaglandin pessary (gemeprost). Contraception 39 497–502. (10.1016/0010-7824(89)90104-2) 2656087

[bib106] Romieu G Maudelonde T Ulmann A Pujol H Grenier J Cavalie G Khalaf S Rochefort H 1987 The antiprogestin RU486 in advanced breast cancer: preliminary clinical trial. Bulletin du Cancer 74 455–461. 3311238

[bib107] Royal College of Obstetricians and Gynaecologists 2011 The care of women requesting induced abortion [Online]. (available at: https://www.rcog.org.uk/globalassets/documents/guidelines/abortion-guideline_web_1.pdf). Accessed.

[bib108] Royal College of Obstetricians and Gynaecologists 2014 National heavy menstrual bleeding audit [Online], London (available at: https://www.rcog.org.uk/globalassets/documents/guidelines/research--audit/national_hmb_audit_final_report_july_2014.pdf). Accessed.

[bib109] Savaris RF Groll JM Young SL Demayo FJ Jeong JW Hamilton AE Giudice LC Lessey BA 2011 Progesterone resistance in PCOS endometrium: a microarray analysis in clomiphene citrate-treated and artificial menstrual cycles. Journal of Clinical Endocrinology and Metabolism 96 1737–1746. (10.1210/jc.2010-2600) 21411543 PMC3100753

[bib110] Schaff EA Fielding SL Westhoff C Ellertson C Eisinger SH Stadalius LS Fuller L 2000 Vaginal misoprostol administered 1, 2, or 3 days after mifepristone for early medical abortion: A randomized trial. JAMA 284 1948–1953. (10.1001/jama.284.15.1948) 11035891

[bib111] Schoep ME Nieboer TE Van Der Zanden M Braat DDM Nap AW 2019 The impact of menstrual symptoms on everyday life: a survey among 42,879 women. American Journal of Obstetrics and Gynecology 220 569.e1–569.e7. (10.1016/j.ajog.2019.02.048) 30885768

[bib113] Schultze-Mosgau MH Schuett B Hafner FT Zollmann F Kaiser A Hoechel J Rohde B 2017 Pharmacokinetics and safety of the selective progesterone receptor modulator vilaprisan in healthy postmenopausal women. International Journal of Clinical Pharmacology and Therapeutics 55 16–24. (10.5414/CP202756) 27841155

[bib112] Schultze-Mosgau MH Höchel J Prien O Zimmermann T Brooks A Bush J Rottmann A 2018 Characterization of the pharmacokinetics of Vilaprisan: bioavailability, excretion, biotransformation, and drug–drug interaction potential. Clinical Pharmacokinetics 57 1001–1015. (10.1007/s40262-017-0607-4) 29330782 PMC6028879

[bib114] Schütt B Kaiser A Schultze-Mosgau MH Seitz C Bell D Koch M Rohde B 2016 Pharmacodynamics and safety of the novel selective progesterone receptor modulator vilaprisan: a double-blind, randomized, placebo-controlled phase 1 trial in healthy women. Human Reproduction 31 1703–1712. (10.1093/humrep/dew140) 27288475

[bib115] Schütt B Schultze-Mosgau MH Draeger C Chang X Löwen S Kaiser A Rohde B 2018 Effect of the novel selective progesterone receptor modulator vilaprisan on ovarian activity in healthy women. Journal of Clinical Pharmacology 58 228–239. (10.1002/jcph.998) 28940451 PMC5813164

[bib116] Shapley M Jordan K Croft PR 2004 An epidemiological survey of symptoms of menstrual loss in the community. British Journal of General Practice 54 359–363. 15113519 PMC1266170

[bib118] Shen Q Hua Y Jiang W Zhang W Chen M Zhu X 2013 Effects of mifepristone on uterine leiomyoma in premenopausal women: a meta-analysis. Fertility and Sterility 100 1722.e1–1726.e1. (10.1016/j.fertnstert.2013.08.039) 24094421

[bib117] Shen J Showell Y Chen EK Cheng L 2019 Interventions for emergency contraception. Cochrane Database of Systematic Reviews 1 CD001324. 30661244 10.1002/14651858.CD001324.pub6 PMC7055045

[bib119] Simon JA Catherino W Segars JH Blakesley RE Chan A Sniukiene V al-hendy A 2018 Ulipristal acetate for treatment of symptomatic uterine leiomyomas: a randomized controlled trial. Obstetrics and Gynecology 131 431–439. (10.1097/AOG.0000000000002462) 29420395 PMC7968372

[bib120] Spitz IM Croxatto HB Robbins A 1996 Antiprogestins: mechanism of action and contraceptive potential. Annual Review of Pharmacology and Toxicology 36 47–81. (10.1146/annurev.pa.36.040196.000403) 8725382

[bib121] Stratton P Hartog B Hajizadeh N Piquion J Sutherland D Merino M Lee YJ Nieman LK 2000 A single mid-follicular dose of CDB-2914, a new antiprogestin, inhibits folliculogenesis and endometrial differentiation in normally cycling women. Human Reproduction 15 1092–1099. (10.1093/humrep/15.5.1092) 10783359

[bib122] Stratton P Levens ED Hartog B Piquion J Wei Q Merino M Nieman LK 2010 Endometrial effects of a single early luteal dose of the selective progesterone receptor modulator CDB-2914. Fertility and Sterility 93 2035–2041. (10.1016/j.fertnstert.2008.12.057) 19200989 PMC2911236

[bib123] Task Force on Postovulatory Methods of Fertility Regulation 1999 Comparison of three single doses of mifepristone as emergency contraception: a randomised trial. Task Force on Postovulatory Methods of Fertility Regulation. Lancet 353 697–702. (10.1016/S0140-6736(98)07190-6) 10073511

[bib124] Taylor HS Bagot C Kardana A Olive D Arici A 1999 HOX gene expression is altered in the endometrium of women with endometriosis. Human Reproduction 14 1328–1331. (10.1093/humrep/14.5.1328) 10325287

[bib125] The Faculty of Sexual & Reproductive Healthcare 2017 Emergency contraception [Online]. (available at: https://www.fsrh.org/standards-and-guidance/documents/ceu-clinical-guidance-emergency-contraception-march-2017/). Accessed.

[bib126] Tinelli A Hurst BS Hudelist G Tsin DA Stark M Mettler L Guido M Malvasi A 2012a Laparoscopic myomectomy focusing on the myoma pseudocapsule: technical and outcome reports. Human Reproduction 27 427–435. (10.1093/humrep/der369) 22095838

[bib127] Tinelli A Malvasi A Hurst BS Tsin DA Davila F Dominguez G Dell’edera D Cavallotti C Negro R Gustapane S, ***et al*** 2012b Surgical management of neurovascular bundle in uterine fibroid pseudocapsule. Journal of the Society of Laparoendoscopic Surgeons 16 119–129. (10.4293/108680812X13291597716302) 22906340 PMC3407432

[bib128] Tristan M Orozco LJ Steed A Ramírez-Morera A Stone P 2012 Mifepristone for uterine fibroids. Cochrane Database of Systematic Reviews CD007687 (10.1002/14651858.CD007687.pub2) 22895965 PMC8212859

[bib129] Wagenfeld A Bone W Schwede W Fritsch M Fischer OM Moeller C 2013 BAY 1002670: a novel, highly potent and selective progesterone receptor modulator for gynaecological therapies. Human Reproduction 28 2253–2264. (10.1093/humrep/det247) 23739217

[bib130] Warner PE Critchley HO Lumsden MA Campbell-Brown M Douglas A Murray GD 2004 Menorrhagia I: measured blood loss, clinical features, and outcome in women with heavy periods: a survey with follow-up data. American Journal of Obstetrics and Gynecology 190 1216–1223. (10.1016/j.ajog.2003.11.015) 15167821

[bib131] Whitaker LH Murray AA Matthews R Shaw G Williams AR Saunders PT Critchley HO 2017 Selective progesterone receptor modulator (SPRM) ulipristal acetate (UPA) and its effects on the human endometrium. Human Reproduction 32 531–543. (10.1093/humrep/dew359) 28130434 PMC5400066

[bib132] Williams AR Bergeron C Barlow DH Ferenczy A 2012 Endometrial morphology after treatment of uterine fibroids with the selective progesterone receptor modulator, ulipristal acetate. International Journal of Gynecological Pathology 31 556–569. (10.1097/PGP.0b013e318251035b) 23018219

[bib134] Xu Q Takekida S Ohara N Chen W Sitruk-Ware R Johansson ED Maruo T 2005 Progesterone receptor modulator CDB-2914 down-regulates proliferative cell nuclear antigen and Bcl-2 protein expression and up-regulates caspase-3 and poly(adenosine 5′-diphosphate-ribose) polymerase expression in cultured human uterine leiomyoma cells. Journal of Clinical Endocrinology and Metabolism 90 953–961. (10.1210/jc.2004-1569) 15572421

[bib133] Xu Q Ohara N Liu J Amano M Sitruk-Ware R Yoshida S Maruo T 2008 Progesterone receptor modulator CDB-2914 induces extracellular matrix metalloproteinase inducer in cultured human uterine leiomyoma cells. Molecular Human Reproduction 14 181–191. (10.1093/molehr/gan004) 18216291

[bib135] Yuzpe AA Lancee WJ 1977 Ethinylestradiol and dl-norgestrel as a postcoital contraceptive. Fertility and Sterility 28 932–936. (10.1016/S0015-0282(16)42793-7) 892044

